# Synthesis and crystal structure of racemic (R*,R*)-2,2′-(1,4-phenyl­ene)bis­(3-phenyl-2,3,5,6-tetra­hydro-4*H*-1,3-thia­zin-4-one)

**DOI:** 10.1107/S2056989021011592

**Published:** 2021-11-09

**Authors:** Hemant P. Yennawar, Joseph J. Medica, Lee J. Silverberg

**Affiliations:** a Pennsylvania State University, 8 Althouse Laboratory, University Park, PA 16802, USA; b Pennsylvania State University, Schuylkill Campus, 200 University Drive, Schuylkill Haven, PA 17972, USA

**Keywords:** bis-heterocycle, twisted boat pucker, half-chair pucker and 1,3-thia­zin-4-one, C—H⋯S hydrogen bonding, crystal structure

## Abstract

In the racemic title compound, C_26_H_24_N_2_O_2_S_2_, one of the thia­zine rings shows a twisted boat conformation (*Q* = 0.743 Å, θ = 92.1°) and the other a half-chair puckering (*Q* = 0.669 Å, θ = 54.3°). The terminal phenyl rings are almost parallel to each other [dihedral angle 21.71 (10)°]. Both of these rings are orthogonal to the central phenyl ring, subtending a dihedral angle of about 78° in each case. The extended structure is consolidated by C—H⋯O and C—H⋯S hydrogen bonds as well as aromatic ring inter­actions of parallel-displaced and T-type. The mol­ecule has approximate *C*2 local symmetry but this is not carried over to its three-dimensional structure or the inter­molecular inter­actions.

## Abstract 2

The synthesis and crystal structure of the racemic title compound C_26_H_24_N_2_O_2_S_2_ with two stereocenters is reported.

## Chemical context

Bis-heterocyclic compounds exhibit a variety of biological activities (Shaker, 2012 ▸). 2,2′-(1,4-Phenyl­ene)-bis-(3-phenyl-1,3-thia­zolidin-4-one), a phenyl­ene-bridged bis-(1,3-thia­zolidin-4-one) in which the bridging benzene ring is connected to C2 of each five-membered heterocycle, has been reported by three groups (Martani, 1956 ▸; Shaker, 1999 ▸; Mohammadi *et al.*, 2020 ▸). 2,2′-(1,4-Phenyl­ene)-bis-(3-(4-fluoro­phen­yl)-1,3-thia­zolidin-4-one) has shown good anti­fungal activity (Abdel-Rahman & Ali, 2013 ▸). The only report of a phenyl­ene-bridged bis-(1,3-thia­zin-4-one) in which the bridging benzene ring is connected to C2 of each six-membered heterocycle is of two unsaturated derivatives of 2,2′-(1,4-phenyl­ene)-bis-(3,4-di­hydro-2*H*-1,3-thia­zin-4-one) (Shaker *et al.*, 2010 ▸). We have previously reported the synthesis and crystal structure of saturated *meso*-3,3′-(1,4-phenyl­ene)-bis-(2-phenyl-2,3,5,6-tetra­hydro-4*H*-1,3-thia­zin-4-one), in which the bridging benzene ring is connected to the nitro­gen atom of each heterocycle (Yennawar, Moyer & Silverburg, 2018 ▸). Herein, we report the synthesis and crystal structure of the racemic title compound, C_26_H_24_N_2_O_2_S_2_. 
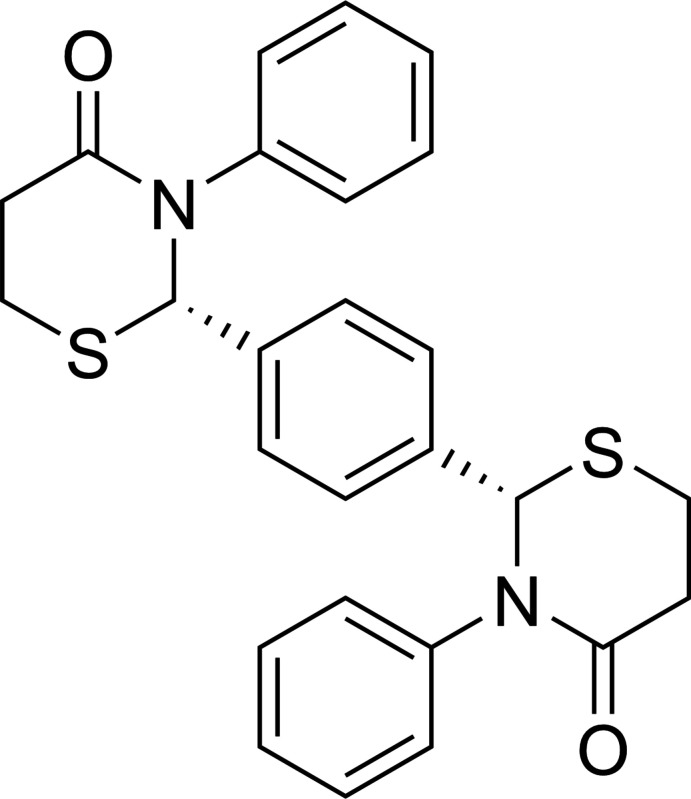


## Structural commentary

**Figure 1:**
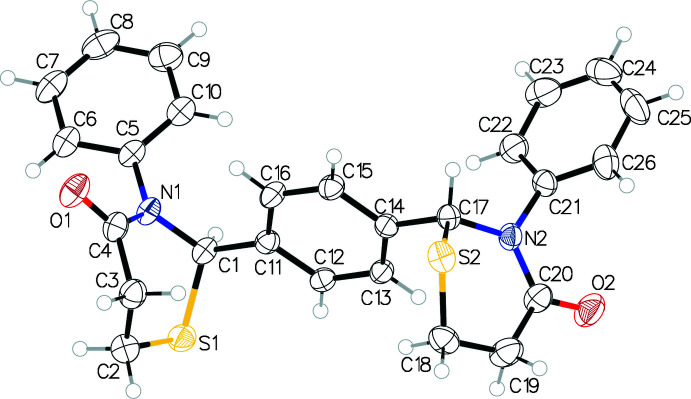
Displacement ellipsoid drawing at a 50% probability level of the title compound.

**Figure 2:**
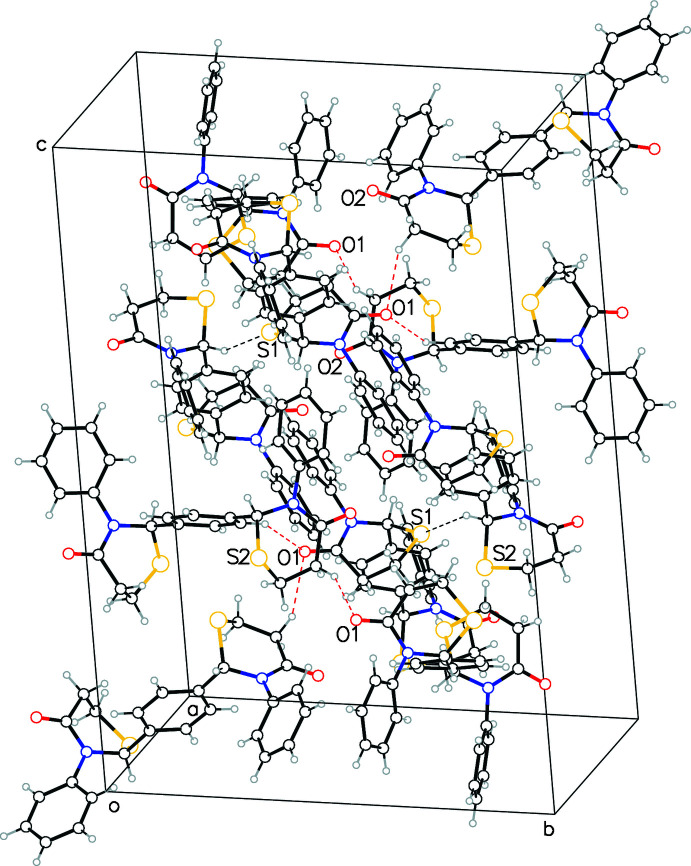
Crystal packing diagram showing C—H⋯O bonds as red dashed lines and C—H⋯S as black dashed lines.

The title compound has three phenyl rings (C5–C10, C11–C16 and C21–C26) alternating with two 1,3-thia­zine rings, C1–C4/N1/S1 and C17–C20/N2/S2 (Fig. 1 ▸). In the arbitrarily chosen asymmetric mol­ecule, atoms C1 and C17 both have an *R* configuration, but crystal symmetry generates a racemic *R,R* and *S,S* mixture. The terminal C5 and C21 phenyl rings are approximately parallel to each other with the inter­planar angle being 21.71 (10)°. Each of these ‘bookend’ rings is orthogonal to the central phenyl ring subtending dihedral angles of 78.50 (9) (C5/C11 rings) and 78.80 (9)° (C11/C21 rings). The thia­zine ring containing atom S1 exhibits a twisted-boat pucker (*Q* = 0.743 Å, θ = 92.1°) while the ring containing S2 has a half-chair pucker (*Q* = 0.669 Å, θ = 54.3°) with atom S2 displaced from the plane defined by the remaining five atoms of the ring, by about 0.968 Å. Neither of these puckers are of the most favored type. Despite the different conformations of the heterocyclic rings, the mol­ecule possesses approximate local *C*2 symmetry with an r.m.s. deviation for the overlapping halves of the mol­ecule of 0.261 Å but this local symmetry does not coincide with any crystallographic symmetry in the lattice. Consequently there is asymmetry in the inter­molecular inter­actions (Fig. 2 ▸).

## Supra­molecular features

**Figure 3:**
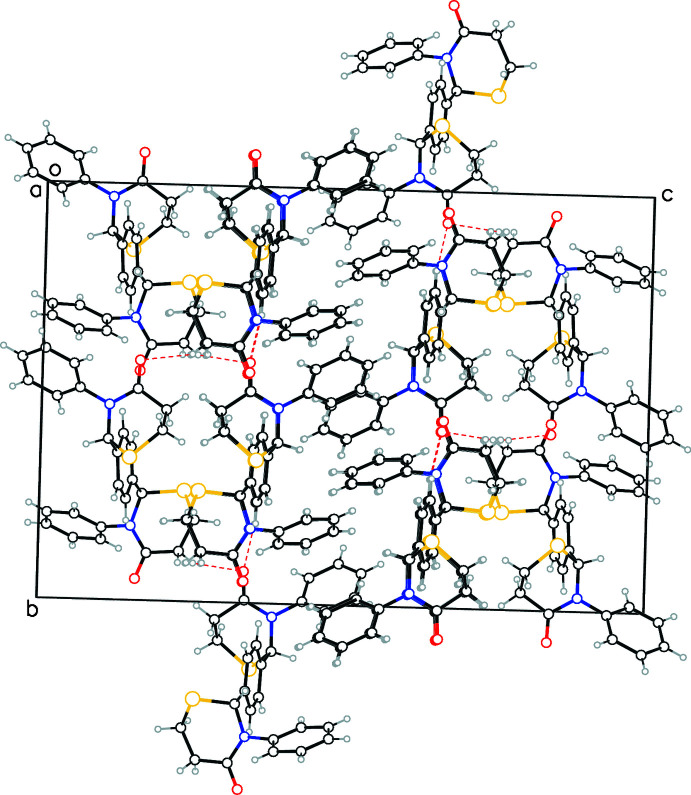
Crystal packing viewed down the *a*-axis direction showing alternate layering of aromatic rings and thia­zine rings in the *c*-axis direction.

The surface of the mol­ecule is dominated by hydro­phobic regions with three phenyl rings alternating with two thia­zine rings. The extended structure seems to be primarily a result of hydro­phobic van der Waals inter­actions, further assisted by aromatic–aromatic inter­actions of parallel-displaced and T-type. Of the two sulfur and two oxygen atoms in each mol­ecule, only one of each (O1 and S1) act as acceptors for C—H⋯O and C—H⋯S type inter­molecular inter­actions (Table 1 ▸). The donor carbon atoms (C13, C17 and C19) are either members of the central phenyl ring or the other thia­zine ring (containing O2 and S2). Thus although the mol­ecule is chemically symmetric and the structure contains both enanti­omers, the packing shows asymmetry in the inter­actions. A view down the *a*-axis direction (Fig. 3 ▸) shows layers of the aromatic rings and thia­zine rings alternating with each other along the *c*-axis direction. It is worth noting the C—H⋯S inter­action observed here is a hydrogen bond between non-traditional donor and acceptor atoms. Ghosh *et al.* (2020 ▸) have recently presented experimental and theoretical analyses of such inter­actions, and state that these type of inter­actions ‘exhibit all the characteristics of conventional hydrogen bonds’.

## Database survey

A structure search was done in Scifinder, and a text search (‘1,3-thia­zin-4-one’) was performed in the Cambridge Structural Database (Groom *et al.*, 2016 ▸; accessed October, 2021). Of the bis-heterocyles mentioned in the *Chemical context* section above, only our prior paper reports a crystal structure (Yennawar, Moyer & Silverburg, 2018 ▸). Crystal structures of monocyclic 2,3,5,6-tetra­hydro-1,3-thia­zin-4-ones have been reported for four 2,3-diaryl-1,3-thia­zin-4-ones (Yennawar & Silverberg, 2014 ▸; Yennawar *et al.*, 2015 ▸; Yennawar, Bradley *et al.*, 2018 ▸), for a bicyclic aza­sugar 2,3-fused 1,3-thia­zin-4-one (Li *et al.*, 2012 ▸) and a 2,2-dialkyl-1,3-thia­zinone (Capps *et al.*, 1985 ▸). The thia­zine rings in these structures exhibit varied puckering. There is a pucker ‘between half-chair and envelope’ in *meso*-3,3′-(1,4-phenyl­ene)bis­(2-phenyl-2,3,5,6-tetra­hydro-4*H*-1,3-thia­zin-4-one (Yennawar, Moyer & Silverberg 2018 ▸), an envelope in 2,3-diphenyl-2,3,5,6-tetra­hydro-4*H*-1,3-thia­zin-4-one (Yennawar & Silverberg, 2014 ▸), envelopes in (2*S*)-2-(3-nitro­phen­yl)-3-phenyl-2,3,5,6-tetra­hydro-4*H*-1,3-thia­zin-4-one and *rac*-2-(4-nitro­phen­yl)-3-phenyl-2,3,5,6-tetra­hydro-4*H*-1,3-thia­zin-4-one (Yennawar, Bradley *et al.*, 2018 ▸), a boat and a half-chair in *N*-[(2*S*,5*R*)-4-oxo-2,3-diphenyl-1,3-thia­zinan-5-yl]acetamide (Yennawar *et al.*, 2015 ▸), a half-chair in (7*R*,8*R*,9*R*,9a*R*)-7,8,9-tri­hydroxy­hexa­hydro-4*H*,6*H*-pyrido[2,1-*b*][1,3]thia­zin-4-one (Li *et al.*, 2012 ▸), and a half-chair and a chair in methyl (2*R*,6*R*)-6-meth­oxy-4-oxo-2-(prop-1-en-2-yl)-1,3-thia­zinane-2-carboxyl­ate (Capps *et al.*, 1985 ▸).

## Synthesis and crystallization

A two-necked 25 ml round-bottom flask was oven-dried, cooled under N_2_, and charged with a stir bar. *N*,*N*′-(Benzene-1,4-diyldi­methylyl­idene)dianiline (0.572 g, 3.00 mmol) and 3-mercaptopropionic acid (0.7432 g, 7.50 mmol) were added. 2-Methyl­tetra­hydro­furan (2.3 ml) was added and the solution was stirred. Pyridine (2.4 ml, 30 mmol) was added. Finally, 2,4,6-tripropyl-1,3,5,2,4,6-trioxatri­phospho­rinane-2,4,6-trioxide (T3P) in 2-methyl­tetra­hydro­furan (50 weight percent; 9.2 ml, 15 mmol) was added. The reaction was stirred at room temperature and followed by TLC, then poured into a separatory funnel with di­chloro­methane (20 ml). The mixture was washed with water (10 ml). The aqueous fraction was then extracted twice with di­chloro­methane (10 ml each). The organics were combined and washed with saturated sodium bicarbonate (10 ml) and then saturated sodium chloride (10 ml). The organic fraction was dried over sodium sulfate and concentrated under vacuum to give a pale yellow crude solid. Recrystallization from ethanol solution gave two crops of off-white solid (0.4715 g and 0.1087 g, total 0.5802 g, 42%). m.p. 476.3–483.7 K (decomp.). Crystals suitable for X-ray analysis were grown by slow evaporation from ethanol solution.

## Refinement

Crystal data, data collection and structure refinement details are summarized in Table 2 ▸.

## Supplementary Material

Crystal structure: contains datablock(s) I. DOI: 10.1107/S2056989021011592/hb7986sup1.cif

Supporting information file. DOI: 10.1107/S2056989021011592/hb7986Isup3.cml

Structure factors: contains datablock(s) I. DOI: 10.1107/S2056989021011592/hb7986Isup4.hkl

CCDC reference: 2119921

Additional supporting information:  crystallographic information; 3D view; checkCIF report

## Figures and Tables

**Table 1:** Hydrogen-bond geometry (Å, °)

*D*—H⋯*A*	*D*—H	H⋯*A*	*D*⋯*A*	*D*—H⋯*A*
C19—H19*A*⋯O1^i^	0.97	2.46	3.353 (3)	153
C17—H17⋯S1^ii^	0.98	2.62	3.5094 (19)	151
C13—H13⋯O1^iii^	0.93	2.52	3.280 (2)	139

Symmetry codes: (i) -x+1, y+{\script{1\over 2}}, -z+{\script{1\over 2}}; (ii) x+1, y, z; (iii) -x+{\script{1\over 2}}, y+{\script{1\over 2}}, z.

**Table 2:** Experimental details

Crystal data	
Chemical formula	C_26_H_24_N_2_O_2_S_2_
*M*_r_	460.59
Crystal system, space group	Orthorhombic, *P* *b* *c* *a*
Temperature (K)	173
*a*, *b*, *c* (Å)	9.6963 (3), 17.6307 (4), 25.7044 (6)
*V* (Å^3^)	4394.22 (19)
*Z*	8
Radiation type	Cu *K*α
μ (mm^−1^)	2.41
Crystal size (mm)	0.34 × 0.30 × 0.16

Data collection	
Diffractometer	ROD, Synergy Custom system, HyPix-Arc 150
Absorption correction	Gaussian (*CrysAlis PRO*; Rigaku OD, 2021 ▸)
*T*_min_, *T*_max_	0.59, 0.81
No. of measured, independent and observed [*I* > 2σ(*I*)] reflections	16911, 4317, 3910
*R*_int_	0.035
(sin θ/λ)_max_ (Å^−1^)	0.627

Refinement	
*R*[*F* ^2^ > 2σ(*F* ^2^)], *wR*(*F* ^2^), *S*	0.040, 0.102, 1.05
No. of reflections	4317
No. of parameters	290
H-atom treatment	H-atom parameters constrained
Δρ_max_, Δρ_min_ (e Å^−3^)	0.34, −0.30

Computer programs: *CrysAlis PRO* (Rigaku OD, 2021 ▸), *CrysAlis PRO* (Rigaku OD, 2021 ▸), *SHELXT2018* (Sheldrick, 2015*a* ▸), *SHELXL2018/3* (Sheldrick, 2015*b* ▸), and *OLEX2* (Dolomanov *et al.*, 2009 ▸).

## supplementary crystallographic information

### Crystal data

**Table:**

C_26_H_24_N_2_O_2_S_2_	*D*_x_ = 1.392 Mg m^−^^3^
*M**_r_* = 460.59	Cu *K*α radiation, λ = 1.54184 Å
Orthorhombic, *P**b**c**a*	Cell parameters from 13210 reflections
*a* = 9.6963 (3) Å	θ = 3.0–74.7°
*b* = 17.6307 (4) Å	µ = 2.41 mm^−^^1^
*c* = 25.7044 (6) Å	*T* = 173 K
*V* = 4394.22 (19) Å^3^	Block, colorless
*Z* = 8	0.34 × 0.30 × 0.16 mm
*F*(000) = 1936	

### Data collection

**Table:**

ROD, Synergy Custom system, HyPix-Arc 150 diffractometer	4317 independent reflections
Radiation source: Rotating-anode X-ray tube	3910 reflections with *I* > 2σ(*I*)
Detector resolution: 10.0000 pixels mm^-1^	*R*_int_ = 0.035
ω scans	θ_max_ = 75.2°, θ_min_ = 3.4°
Absorption correction: gaussian (CrysalisPro; Rigaku OD, 2021)	*h* = −11→10
*T*_min_ = 0.59, *T*_max_ = 0.81	*k* = −21→21
16911 measured reflections	*l* = −31→22

### Refinement

**Table:**

Refinement on *F*^2^	Hydrogen site location: inferred from neighbouring sites
Least-squares matrix: full	H-atom parameters constrained
*R*[*F*^2^ > 2σ(*F*^2^)] = 0.040	*w* = 1/[σ^2^(*F*_o_^2^) + (0.043*P*)^2^ + 2.8735*P*] where *P* = (*F*_o_^2^ + 2*F*_c_^2^)/3
*wR*(*F*^2^) = 0.102	(Δ/σ)_max_ < 0.001
*S* = 1.05	Δρ_max_ = 0.34 e Å^−^^3^
4317 reflections	Δρ_min_ = −0.30 e Å^−^^3^
290 parameters	Extinction correction: SHELXL2018/3 (Sheldrick 2015b), Fc^*^=kFc[1+0.001xFc^2^λ^3^/sin(2θ)]^-1/4^
0 restraints	Extinction coefficient: 0.00067 (6)

### Special details

**Table:**

Geometry. All esds (except the esd in the dihedral angle between two l.s. planes) are estimated using the full covariance matrix. The cell esds are taken into account individually in the estimation of esds in distances, angles and torsion angles; correlations between esds in cell parameters are only used when they are defined by crystal symmetry. An approximate (isotropic) treatment of cell esds is used for estimating esds involving l.s. planes.

### Fractional atomic coordinates and isotropic or equivalent isotropic displacement parameters (Å^2^)

**Table:**

	*x*	*y*	*z*	*U*_iso_*/*U*_eq_	
S1	0.03452 (5)	0.15757 (3)	0.35406 (2)	0.03576 (14)	
S2	0.74104 (5)	0.23378 (3)	0.26135 (2)	0.03303 (14)	
O1	0.17980 (17)	−0.07596 (8)	0.34004 (5)	0.0405 (4)	
O2	0.73414 (19)	0.44837 (8)	0.33902 (6)	0.0498 (4)	
N1	0.17158 (16)	0.03164 (8)	0.38871 (6)	0.0285 (3)	
N2	0.74004 (16)	0.32127 (9)	0.34850 (6)	0.0282 (3)	
C1	0.17917 (19)	0.11440 (10)	0.38934 (7)	0.0283 (4)	
H1	0.166679	0.129095	0.425801	0.034*	
C2	0.0161 (2)	0.09064 (12)	0.30065 (8)	0.0396 (5)	
H2A	−0.063280	0.058472	0.306954	0.048*	
H2B	0.000097	0.118287	0.268585	0.048*	
C3	0.1439 (2)	0.04154 (11)	0.29466 (7)	0.0351 (4)	
H3A	0.133360	0.009144	0.264420	0.042*	
H3B	0.223572	0.073762	0.289008	0.042*	
C4	0.1679 (2)	−0.00726 (11)	0.34247 (7)	0.0315 (4)	
C5	0.1762 (2)	−0.00729 (10)	0.43785 (7)	0.0289 (4)	
C6	0.0811 (2)	−0.06443 (10)	0.44850 (8)	0.0330 (4)	
H6	0.015814	−0.078494	0.423799	0.040*	
C7	0.0854 (2)	−0.09993 (11)	0.49658 (8)	0.0401 (5)	
H7	0.023888	−0.139005	0.503783	0.048*	
C8	0.1797 (3)	−0.07817 (12)	0.53393 (8)	0.0441 (5)	
H8	0.181054	−0.102412	0.566059	0.053*	
C9	0.2717 (2)	−0.02062 (12)	0.52368 (8)	0.0417 (5)	
H9	0.334019	−0.005143	0.549063	0.050*	
C10	0.2710 (2)	0.01427 (11)	0.47510 (8)	0.0351 (4)	
H10	0.334578	0.052247	0.467703	0.042*	
C11	0.31846 (18)	0.14655 (10)	0.37342 (6)	0.0257 (4)	
C12	0.33492 (19)	0.22501 (10)	0.37044 (7)	0.0262 (4)	
H12	0.259894	0.256443	0.377028	0.031*	
C13	0.46121 (18)	0.25695 (10)	0.35783 (6)	0.0262 (4)	
H13	0.470079	0.309434	0.356459	0.031*	
C14	0.57488 (19)	0.21130 (10)	0.34719 (6)	0.0251 (4)	
C15	0.5598 (2)	0.13267 (10)	0.35063 (7)	0.0289 (4)	
H15	0.634864	0.101322	0.343891	0.035*	
C16	0.4333 (2)	0.10086 (10)	0.36410 (7)	0.0302 (4)	
H16	0.425202	0.048454	0.366908	0.036*	
C17	0.71351 (19)	0.24304 (10)	0.33082 (7)	0.0267 (4)	
H17	0.783403	0.211186	0.347587	0.032*	
C18	0.6139 (2)	0.30351 (11)	0.24347 (7)	0.0362 (4)	
H18A	0.612491	0.308869	0.205912	0.043*	
H18B	0.523504	0.286260	0.254475	0.043*	
C19	0.6438 (3)	0.37990 (12)	0.26799 (8)	0.0437 (5)	
H19A	0.703209	0.407306	0.244148	0.052*	
H19B	0.557206	0.407369	0.269383	0.052*	
C20	0.7086 (2)	0.38557 (11)	0.32129 (7)	0.0344 (4)	
C21	0.81722 (19)	0.32632 (10)	0.39613 (7)	0.0292 (4)	
C22	0.7625 (2)	0.29558 (11)	0.44129 (7)	0.0370 (5)	
H22	0.672942	0.276680	0.441353	0.044*	
C23	0.8408 (3)	0.29297 (12)	0.48625 (8)	0.0495 (6)	
H23	0.804317	0.271700	0.516364	0.059*	
C24	0.9725 (3)	0.32179 (13)	0.48645 (10)	0.0544 (7)	
H24	1.024965	0.320241	0.516726	0.065*	
C25	1.0268 (3)	0.35300 (14)	0.44176 (11)	0.0533 (6)	
H25	1.115824	0.372603	0.442092	0.064*	
C26	0.9499 (2)	0.35544 (12)	0.39634 (9)	0.0408 (5)	
H26	0.987103	0.376435	0.366260	0.049*	

### Atomic displacement parameters (Å^2^)

**Table:**

	*U*^11^	*U*^22^	*U*^33^	*U*^12^	*U*^13^	*U*^23^
S1	0.0283 (2)	0.0316 (2)	0.0474 (3)	0.00307 (19)	0.0025 (2)	0.0052 (2)
S2	0.0318 (3)	0.0391 (3)	0.0282 (2)	−0.0025 (2)	0.00399 (18)	−0.00733 (18)
O1	0.0579 (10)	0.0262 (7)	0.0374 (7)	−0.0022 (7)	0.0097 (7)	−0.0020 (6)
O2	0.0725 (11)	0.0269 (7)	0.0501 (9)	−0.0046 (7)	−0.0080 (8)	−0.0010 (6)
N1	0.0342 (8)	0.0225 (7)	0.0286 (7)	−0.0038 (6)	0.0052 (6)	0.0026 (6)
N2	0.0314 (8)	0.0254 (8)	0.0277 (7)	−0.0048 (6)	−0.0017 (6)	−0.0021 (6)
C1	0.0321 (10)	0.0225 (8)	0.0304 (9)	−0.0007 (7)	0.0045 (8)	0.0019 (7)
C2	0.0385 (11)	0.0382 (11)	0.0421 (11)	−0.0035 (9)	−0.0049 (9)	0.0039 (9)
C3	0.0407 (11)	0.0331 (10)	0.0313 (10)	−0.0044 (8)	0.0014 (8)	0.0012 (8)
C4	0.0329 (10)	0.0285 (9)	0.0330 (9)	−0.0039 (8)	0.0064 (8)	0.0002 (8)
C5	0.0346 (10)	0.0229 (8)	0.0290 (9)	0.0015 (7)	0.0058 (8)	0.0015 (7)
C6	0.0379 (10)	0.0261 (9)	0.0351 (10)	−0.0013 (8)	0.0095 (8)	−0.0002 (8)
C7	0.0504 (13)	0.0279 (9)	0.0420 (11)	0.0014 (9)	0.0165 (10)	0.0067 (8)
C8	0.0612 (15)	0.0378 (11)	0.0332 (10)	0.0099 (10)	0.0122 (10)	0.0095 (9)
C9	0.0518 (13)	0.0408 (11)	0.0326 (10)	0.0060 (10)	−0.0011 (9)	−0.0002 (9)
C10	0.0400 (11)	0.0298 (9)	0.0356 (10)	−0.0005 (8)	0.0029 (8)	0.0022 (8)
C11	0.0288 (9)	0.0252 (8)	0.0231 (8)	−0.0007 (7)	0.0004 (7)	0.0017 (7)
C12	0.0288 (9)	0.0234 (8)	0.0265 (8)	0.0026 (7)	0.0007 (7)	0.0001 (7)
C13	0.0305 (9)	0.0220 (8)	0.0262 (8)	−0.0010 (7)	−0.0015 (7)	0.0009 (7)
C14	0.0280 (9)	0.0256 (9)	0.0219 (8)	−0.0018 (7)	−0.0023 (7)	−0.0003 (6)
C15	0.0294 (9)	0.0241 (9)	0.0332 (9)	0.0030 (7)	0.0014 (7)	−0.0005 (7)
C16	0.0341 (10)	0.0208 (8)	0.0356 (9)	0.0009 (7)	0.0033 (8)	0.0023 (7)
C17	0.0281 (9)	0.0252 (8)	0.0267 (8)	−0.0015 (7)	−0.0022 (7)	−0.0027 (7)
C18	0.0369 (11)	0.0454 (11)	0.0264 (9)	−0.0007 (9)	−0.0027 (8)	0.0000 (8)
C19	0.0611 (15)	0.0363 (11)	0.0338 (10)	0.0019 (10)	−0.0029 (10)	0.0048 (8)
C20	0.0409 (11)	0.0306 (10)	0.0319 (9)	−0.0011 (8)	0.0023 (8)	0.0012 (8)
C21	0.0311 (9)	0.0266 (8)	0.0301 (9)	0.0000 (7)	−0.0038 (8)	−0.0064 (7)
C22	0.0467 (12)	0.0331 (10)	0.0312 (10)	−0.0020 (9)	−0.0043 (9)	−0.0030 (8)
C23	0.0799 (18)	0.0361 (11)	0.0324 (11)	0.0070 (11)	−0.0132 (11)	−0.0047 (9)
C24	0.0692 (17)	0.0415 (12)	0.0526 (14)	0.0158 (12)	−0.0321 (13)	−0.0159 (11)
C25	0.0395 (12)	0.0482 (13)	0.0723 (16)	0.0015 (10)	−0.0205 (12)	−0.0260 (12)
C26	0.0372 (11)	0.0368 (10)	0.0485 (12)	−0.0057 (9)	0.0006 (9)	−0.0121 (9)

### Geometric parameters (Å, º)

**Table:**

S1—C2	1.819 (2)	C10—H10	0.9300
S1—C1	1.8355 (19)	C11—C12	1.395 (2)
S2—C18	1.801 (2)	C11—C16	1.395 (2)
S2—C17	1.8130 (18)	C12—C13	1.386 (2)
O1—C4	1.218 (2)	C12—H12	0.9300
O2—C20	1.223 (2)	C13—C14	1.392 (3)
N1—C4	1.373 (2)	C13—H13	0.9300
N1—C5	1.438 (2)	C14—C15	1.397 (2)
N1—C1	1.461 (2)	C14—C17	1.516 (2)
N2—C20	1.367 (2)	C15—C16	1.393 (3)
N2—C21	1.438 (2)	C15—H15	0.9300
N2—C17	1.475 (2)	C16—H16	0.9300
C1—C11	1.521 (2)	C17—H17	0.9800
C1—H1	0.9800	C18—C19	1.515 (3)
C2—C3	1.519 (3)	C18—H18A	0.9700
C2—H2A	0.9700	C18—H18B	0.9700
C2—H2B	0.9700	C19—C20	1.510 (3)
C3—C4	1.518 (3)	C19—H19A	0.9700
C3—H3A	0.9700	C19—H19B	0.9700
C3—H3B	0.9700	C21—C26	1.385 (3)
C5—C10	1.380 (3)	C21—C22	1.387 (3)
C5—C6	1.393 (3)	C22—C23	1.384 (3)
C6—C7	1.386 (3)	C22—H22	0.9300
C6—H6	0.9300	C23—C24	1.374 (4)
C7—C8	1.380 (3)	C23—H23	0.9300
C7—H7	0.9300	C24—C25	1.378 (4)
C8—C9	1.377 (3)	C24—H24	0.9300
C8—H8	0.9300	C25—C26	1.386 (3)
C9—C10	1.392 (3)	C25—H25	0.9300
C9—H9	0.9300	C26—H26	0.9300
			
C2—S1—C1	100.30 (9)	C11—C12—H12	119.4
C18—S2—C17	95.12 (9)	C12—C13—C14	120.70 (16)
C4—N1—C5	121.52 (15)	C12—C13—H13	119.6
C4—N1—C1	120.65 (15)	C14—C13—H13	119.6
C5—N1—C1	117.73 (14)	C13—C14—C15	118.60 (17)
C20—N2—C21	120.05 (15)	C13—C14—C17	122.92 (16)
C20—N2—C17	125.39 (15)	C15—C14—C17	118.47 (16)
C21—N2—C17	114.27 (14)	C16—C15—C14	120.49 (17)
N1—C1—C11	114.46 (15)	C16—C15—H15	119.8
N1—C1—S1	111.73 (13)	C14—C15—H15	119.8
C11—C1—S1	113.03 (12)	C15—C16—C11	120.88 (16)
N1—C1—H1	105.6	C15—C16—H16	119.6
C11—C1—H1	105.6	C11—C16—H16	119.6
S1—C1—H1	105.6	N2—C17—C14	114.48 (15)
C3—C2—S1	111.48 (14)	N2—C17—S2	111.22 (12)
C3—C2—H2A	109.3	C14—C17—S2	111.76 (12)
S1—C2—H2A	109.3	N2—C17—H17	106.2
C3—C2—H2B	109.3	C14—C17—H17	106.2
S1—C2—H2B	109.3	S2—C17—H17	106.2
H2A—C2—H2B	108.0	C19—C18—S2	111.69 (15)
C4—C3—C2	111.48 (16)	C19—C18—H18A	109.3
C4—C3—H3A	109.3	S2—C18—H18A	109.3
C2—C3—H3A	109.3	C19—C18—H18B	109.3
C4—C3—H3B	109.3	S2—C18—H18B	109.3
C2—C3—H3B	109.3	H18A—C18—H18B	107.9
H3A—C3—H3B	108.0	C20—C19—C18	121.05 (17)
O1—C4—N1	122.58 (17)	C20—C19—H19A	107.1
O1—C4—C3	122.46 (17)	C18—C19—H19A	107.1
N1—C4—C3	114.94 (16)	C20—C19—H19B	107.1
C10—C5—C6	120.24 (18)	C18—C19—H19B	107.1
C10—C5—N1	119.90 (16)	H19A—C19—H19B	106.8
C6—C5—N1	119.81 (17)	O2—C20—N2	121.00 (18)
C7—C6—C5	118.79 (19)	O2—C20—C19	118.84 (18)
C7—C6—H6	120.6	N2—C20—C19	120.15 (17)
C5—C6—H6	120.6	C26—C21—C22	119.81 (19)
C8—C7—C6	121.00 (19)	C26—C21—N2	120.64 (18)
C8—C7—H7	119.5	C22—C21—N2	119.31 (17)
C6—C7—H7	119.5	C23—C22—C21	120.1 (2)
C9—C8—C7	120.08 (19)	C23—C22—H22	119.9
C9—C8—H8	120.0	C21—C22—H22	119.9
C7—C8—H8	120.0	C24—C23—C22	120.1 (2)
C8—C9—C10	119.6 (2)	C24—C23—H23	120.0
C8—C9—H9	120.2	C22—C23—H23	120.0
C10—C9—H9	120.2	C23—C24—C25	120.0 (2)
C5—C10—C9	120.27 (19)	C23—C24—H24	120.0
C5—C10—H10	119.9	C25—C24—H24	120.0
C9—C10—H10	119.9	C24—C25—C26	120.6 (2)
C12—C11—C16	118.17 (16)	C24—C25—H25	119.7
C12—C11—C1	119.09 (16)	C26—C25—H25	119.7
C16—C11—C1	122.66 (15)	C21—C26—C25	119.4 (2)
C13—C12—C11	121.12 (17)	C21—C26—H26	120.3
C13—C12—H12	119.4	C25—C26—H26	120.3
			
C4—N1—C1—C11	−72.6 (2)	C13—C14—C15—C16	0.4 (3)
C5—N1—C1—C11	103.92 (18)	C17—C14—C15—C16	−178.19 (16)
C4—N1—C1—S1	57.5 (2)	C14—C15—C16—C11	1.2 (3)
C5—N1—C1—S1	−126.00 (15)	C12—C11—C16—C15	−1.9 (3)
C2—S1—C1—N1	−37.28 (14)	C1—C11—C16—C15	−178.66 (16)
C2—S1—C1—C11	93.55 (14)	C20—N2—C17—C14	−90.8 (2)
C1—S1—C2—C3	−17.14 (16)	C21—N2—C17—C14	95.38 (18)
S1—C2—C3—C4	63.06 (19)	C20—N2—C17—S2	37.1 (2)
C5—N1—C4—O1	−6.2 (3)	C21—N2—C17—S2	−136.75 (13)
C1—N1—C4—O1	170.11 (18)	C13—C14—C17—N2	25.7 (2)
C5—N1—C4—C3	172.13 (17)	C15—C14—C17—N2	−155.73 (16)
C1—N1—C4—C3	−11.5 (3)	C13—C14—C17—S2	−101.90 (17)
C2—C3—C4—O1	125.2 (2)	C15—C14—C17—S2	76.68 (18)
C2—C3—C4—N1	−53.2 (2)	C18—S2—C17—N2	−59.40 (14)
C4—N1—C5—C10	132.03 (19)	C18—S2—C17—C14	69.93 (14)
C1—N1—C5—C10	−44.4 (2)	C17—S2—C18—C19	57.69 (16)
C4—N1—C5—C6	−50.7 (3)	S2—C18—C19—C20	−33.8 (3)
C1—N1—C5—C6	132.83 (18)	C21—N2—C20—O2	−6.6 (3)
C10—C5—C6—C7	−1.3 (3)	C17—N2—C20—O2	179.90 (19)
N1—C5—C6—C7	−178.50 (17)	C21—N2—C20—C19	172.13 (18)
C5—C6—C7—C8	1.6 (3)	C17—N2—C20—C19	−1.4 (3)
C6—C7—C8—C9	−0.3 (3)	C18—C19—C20—O2	178.1 (2)
C7—C8—C9—C10	−1.4 (3)	C18—C19—C20—N2	−0.6 (3)
C6—C5—C10—C9	−0.4 (3)	C20—N2—C21—C26	−61.7 (2)
N1—C5—C10—C9	176.87 (18)	C17—N2—C21—C26	112.5 (2)
C8—C9—C10—C5	1.7 (3)	C20—N2—C21—C22	124.0 (2)
N1—C1—C11—C12	177.14 (15)	C17—N2—C21—C22	−61.8 (2)
S1—C1—C11—C12	47.7 (2)	C26—C21—C22—C23	−0.9 (3)
N1—C1—C11—C16	−6.1 (2)	N2—C21—C22—C23	173.43 (18)
S1—C1—C11—C16	−135.58 (15)	C21—C22—C23—C24	0.9 (3)
C16—C11—C12—C13	0.9 (3)	C22—C23—C24—C25	−0.3 (3)
C1—C11—C12—C13	177.79 (16)	C23—C24—C25—C26	−0.2 (3)
C11—C12—C13—C14	0.7 (3)	C22—C21—C26—C25	0.4 (3)
C12—C13—C14—C15	−1.4 (3)	N2—C21—C26—C25	−173.87 (19)
C12—C13—C14—C17	177.14 (15)	C24—C25—C26—C21	0.2 (3)

### Hydrogen-bond geometry (Å, º)

**Table:**

*D*—H···*A*	*D*—H	H···*A*	*D*···*A*	*D*—H···*A*
C19—H19*A*···O1^i^	0.97	2.46	3.353 (3)	153
C17—H17···S1^ii^	0.98	2.62	3.5094 (19)	151
C13—H13···O1^iii^	0.93	2.52	3.280 (2)	139

Symmetry codes: (i) −*x*+1, *y*+1/2, −*z*+1/2; (ii) *x*+1, *y*, *z*; (iii) −*x*+1/2, *y*+1/2, *z*.
